# Lysine Methyltransferase 9 (KMT9) Is an Actionable Target in Muscle-Invasive Bladder Cancer

**DOI:** 10.3390/cancers16081532

**Published:** 2024-04-17

**Authors:** Sainab Totonji, Anna Ramos-Triguero, Dominica Willmann, Manuela Sum, Sylvia Urban, Helena Bauer, Astrid Rieder, Sheng Wang, Holger Greschik, Eric Metzger, Roland Schüle

**Affiliations:** 1 Klinik für Urologie und Zentrale Klinische Forschung, Klinikum der Albert-Ludwigs-Universität Freiburg, 79106 Freiburg, Germany; sainab.totonji@uniklinik-freiburg.de (S.T.);; 2 Deutsches Konsortium für Translationale Krebsforschung, Standort Freiburg, 79106 Freiburg, Germany; 3 CIBSS Centre of Biological Signalling Studies, University of Freiburg, 79104 Freiburg, Germany

**Keywords:** KMT9, lysine methyltransferase, bladder cancer, proliferation, migration, invasion, inhibitor

## Abstract

**Simple Summary:**

The recently identified lysine methyltransferase (KMT) 9 regulates the growth of different types of cancer. While KMT9 was shown to be overexpressed in muscle-invasive bladder cancer (MIBC) tissue samples of patients, a potential functional role of the enzyme in MIBC remains to be clarified. In this study, we show that KMT9 regulates the proliferation, migration, and invasion of various MIBC cell lines as well as the growth of BC tumor organoids and xenografts in mice. Our data provide evidence that tumor cell growth relies on the enzymatic function of KMT9 and that a small-molecule inhibitor of KMT9 impairs BC cell proliferation. These results suggest that KMT9 is a potential novel therapeutic target for MIBC treatment.

**Abstract:**

Novel treatment modalities are imperative for the challenging management of muscle-invasive and metastatic BC to improve patient survival rates. The recently identified KMT9, an obligate heterodimer composed of KMT9α and KMT9β, regulates the growth of various types of tumors such as prostate, lung, and colon cancer. While the overexpression of KMT9α was previously observed to be associated with aggressive basal-like MIBC in an analysis of patients’ tissue samples, a potential functional role of KMT9 in this type of cancer has not been investigated to date. In this study, we show that KMT9 regulates proliferation, migration, and invasion of various MIBC cell lines with different genetic mutations. KMT9α depletion results in the differential expression of genes regulating the cell cycle, cell adhesion, and migration. Differentially expressed genes include oncogenes such as EGFR and AKT1 as well as mediators of cell adhesion or migration such as DAG1 and ITGA6. Reduced cell proliferation upon KMT9α depletion is also observed in *Pten*/*Trp53* knockout bladder tumor organoids, which cannot be rescued with an enzymatically inactive KMT9α mutant. In accordance with the idea that the catalytic activity of KMT9 is required for the control of cellular processes in MIBC, a recently developed small-molecule inhibitor of KMT9 (KMI169) also impairs cancer cell proliferation. Since KMT9α depletion also restricts the growth of xenografts in mice, our data suggest that KMT9 is an actionable novel therapeutic target for the treatment of MIBC.

## 1. Introduction

Bladder cancer (BC) is the predominant malignancy affecting the urinary tract with a global incidence of 573,000 newly diagnosed cases and 213,000 deaths in the year 2020 [1]. The primary histological types of BC are urothelial carcinoma, which is the most common type, squamous cell carcinoma, and adenocarcinoma [2]. Urothelial carcinoma can be divided into non-muscle-invasive (NMI) BC and muscle-invasive (MI) BC based on tumor infiltration into the bladder wall [3]. NMIBC, accounting for approximately 75% of newly diagnosed cases, manifests as either an exophytic papillary tumor limited to the mucosa (stage Ta according to the tumor-node-metastasis (TNM) classification) or the lamina propria (T1), or as a flat high-grade lesion (carcinoma in situ (CIS)) [4]. MIBC is characterized by a malignant extension into the detrusor muscle (T2), perivesical tissue (T3), or surrounding organs such as the prostatic stroma, seminal vesicles, uterus, vagina, or pelvic and abdominal walls (T4) [5]. MIBC can arise as a new case or develop in 10–20% of progressing NMIBC instances. The five-year survival rate for localized MIBC is about 60% and is dropping to below 10% for patients with distant metastases [6].

The most common risk factors for BC include genetic predisposition, tobacco smoking, exposure to chemicals (such as polycyclic aromatic hydrocarbons or aromatic amines), chronic inflammation, and, possibly, changes in the urinary microbiome (urobiome) [7,8,9,10]. Altered genetic or epigenetic pathways, inflammatory processes, and changes in the urobiome are reflected by the occurrence of (potential) biomarkers. Numerous markers tracing genomic, transcriptomic, epigenetic, proteomic, or urobiome changes in urine samples have been identified [11,12,13,14,15]. They may serve as non-invasive methods complementing cystoscopy and tissue biopsy, which to date is the gold standard for BC detection and follow-up [4,16,17]. However, the limitations of available markers such as low specificity and the lack of independent prognostic urine biomarkers currently restrict their clinical use [12], and the approved non-invasive assays for BC diagnosis and follow-up are underutilized [2]. Recently, changes in the urobiome have attracted significant attention, since they may allow to identify novel, non-invasive screening or monitoring tools for BC diagnosis [10,13,14,15]. A detailed understanding of the interplay between changes in the urobiome, bladder tumorigenesis, and inflammatory processes holds promise for the development of next-generation diagnostic or therapeutic options [18,19].

Treatment of NMIBC involves transurethral resection and intravesical therapy with regular cystoscopy due to a high recurrence [16]. In comparison, platinum-based combination systemic therapies like dose-dense methotrexate, vinblastine, adriamycin, cisplatin (ddMVAC), or gemcitabine plus cisplatin, followed by radical cystectomy with lymph node dissection and urine diversion, remain the standard treatment for advanced localized MIBC [2,20,21,22,23,24]. However, chemotherapeutic resistance or ineligibility in up to 50% of patients for cisplatin-based therapy due to comorbidities lead to low long-term survival rates [25]. Alternative approved medications for platinum-resistant or -ineligible patients with locally advanced or metastatic BC include immune checkpoint inhibitors targeting PD-1 or PD-L1, an FGFR inhibitor, and antibody–drug conjugates against Nectin-4 and Trop-2 [26,27,28,29]. Unfortunately, only a minority of patients with advanced BC, benefit significantly from immune checkpoint inhibitors [30,31], and FGFR alterations are only present in a subset of bladder cancer cases [32]. These low success rates highlight the need for new therapeutic agents as monotherapy or combination therapy options.

Generally, targeted therapies are hampered by complications due to the heterogeneous nature of BC, which is characterized by a high prevalence of distinct mutations [33,34,35]. MIBC often exhibits inactivating mutations in critical tumor suppressors like PTEN, TP53, or RB1 [36]. Accordingly, the deletion of *Pten*/*Trp53* was previously shown to cause MIBC in mice [37,38,39]. For initial studies, organoids, which bridge the gap between time-consuming mouse models and traditional 2D cell cultures, have gained traction due to their better replication of in vivo organ function compared to cell monolayers by maintaining cell–cell and cell–extracellular matrix interactions [40,41]. Murine bladder organoids predominantly consist of basal cells [42], which are considered the cells of origin for MIBC and CIS [43].

Recently, we identified lysine methyltransferase (KMT) 9, an obligate heterodimer consisting of KMT9α (also known as N6AMT1) and KMT9β (also known as TRMT112), as a novel target in cancer. KMT9 monomethylates histone H4 at lysine 12 [44] in addition to other, non-histone proteins [45,46,47]. The enzyme controls the growth of various tumor cell lines, including prostate, lung, and colon cancer cells [44,48,49]. The depletion of KMT9 can inhibit tumor cell proliferation via different mechanisms, for example, by affecting the expression of established cell cycle regulators such as CDK1 or BIRC5, which results in increased G0-G1 arrest and the induction of apoptosis in prostate cancer cells [44]. In comparison, in lung cancer cells, the depletion of KMT9 causes non-apoptotic cell death by deregulating the organization of subcellular organelles [48]. In colorectal cancer, a major function of KMT9 besides cell cycle regulation is the maintenance of colorectal cancer stem and the initiation of cell populations [49]. Consequently, the deletion of *Kmt9α* in mice resulted in the downregulation of multiple stem cell markers, notably, *Apcdd1*, *Dach1*, and *Rhobtb3* [49], which are part of the Lgr5 intestinal stem cell signature. Importantly, using a catalytically inactive mutant, we showed that prostate tumor cell proliferation depends on the enzymatic function of KMT9 [44]. Accordingly, we developed a selective small-molecule KMT9 inhibitor (KMI169) with cellular activity and provided evidence that KMT9 inhibition might be a therapeutic option for the treatment of prostate cancer [50].

In this study, we addressed the question whether KMT9 might also be a therapeutic target in BC. We show that KMT9α knockdown reduces the proliferation, migration, and invasion of various MIBC cell lines as well as the growth of tumor organoids and BC xenografts in mice. Accordingly, in transcriptome analyses, we identified gene sets controlled by KMT9, which account for the deregulation of these tumor-promoting processes. Notably, KMT9α depletion reduces the expression of EGFR and its downstream target protein AKT1, which may in part account for the observed proliferative and migratory defects of BC cells. Importantly, tumor cell growth relies on the enzymatic function of KMT9, and its enzymatic inactivation causes the differential expression of proliferation- and migration-related genes. Consequently, the recently reported small-molecule inhibitor of KMT9, KMI169, impairs BC cell proliferation. In summary, our data suggest that KMT9 is an actionable, novel therapeutic target for the treatment of MIBC.

## 2. Methods

### 2.1. Cell Lines

5637 (#HTB-9) and UM-UC-3 (#CRL-1749) cells were obtained from ATCC (American Type Culture Collection, Manassas, VA, USA). CAL-29 (#ACC 515) and VM-CUB-1 (#ACC 400) cells were obtained from DSMZ-German Collection of Microorganisms and Cell Cultures GmbH (Braunschweig, Germany). UM-UC-6 (#08090503) and UM-UC-10 (#08090506) cells were obtained from Merck KGaA (Darmstadt, Germany).

The other cell lines were kindly provided by the following research groups: HT-1376 (#ACC 397) by M. Timmers (Freiburg, Germany); T24 (ATCC #HTB-4), RT-112, and J82 by S. Garczyk (Aachen, Germany); and TCC-SUP-G (#MK5256) and JON by E. Pitt (Leeds, UK).

### 2.2. Cell Culture

CAL-29, VM-CUB-1, and TCC-SUP-G cells were cultured in DMEM (Ref: 11960-044). JON and RT112 cells were cultured in RPMI 1640 (Ref: 31870-025). 5637 cells were cultured in RPMI 1640 (Ref: A10491-01). HT-1376, UM-UC-3, and J82 cells were cultured in EMEM (Ref: 11090-081). UM-UC-6 and UM-UC-10 cells were cultured in EMEM + 1% Non-Essential Amino Acids (NEAA). T24 cells were cultured in ATCC-formulated McCoy’s 5a Modified Medium (#30-2007). All media were supplemented with 10% fetal calf serum, penicillin/streptomycin, plasmocin, and glutamine.

### 2.3. Transfection with siRNA

Cells were transfected with the indicated siRNAs in the presence of DharmaFECT 1 (#T-2001-03, Horizon Discovery, Cambridge, UK) (HT-1376, UM-UC-6, VM-CUB-1, T24, JON, and TCC-SUP-G), DharmaFECT 3 (#T-2003-03, Horizon Discovery, Cambridge, UK) (5637 and UM-UC-10), DharmaFECT 4 (#T-2004-03, Horizon Discovery, Cambridge, UK) (UM-UC-3) or RNAiMAX (#13778150, Thermo Fisher Scientific (Waltham, MA, USA)) (CAL-29, J82, and RT-112); according to the manufacturer’s instruction with a final siRNA concentration of 90 nM (DharmaFECT 1, 3 and 4) or 50 nM (RNAiMAX). The sequences of the siRNAs (Stealth RNAi™ siRNAs; Life Technologies) used in the experiments are as follows: siCtrl: 5′-GAAAGUCCUAGAUCCACACGCAAAU-3′; siKMT9α#1: 5′-ACGCUGUAACAAAGUUCACAUUCAA-3′; and siKMT9α#2: 5′-CACGCUGUAACAAAGUUCACAUUCA-3′.

### 2.4. Cell Proliferation Assay

Cell proliferation was determined using the xCELLigence RTCA system (Roche, Basle, Suizerland). Real-time recording of cell proliferation was started 24 h after transfection with the indicated siRNAs. In EGFR inhibition assays, cells were seeded in a medium containing the indicated concentration of Erlotinib HCI (OSI-774 HCI, Selleck Chemicals GmbH (Cologne, Germany), #S1023). Cells were seeded in 16-well E-plates (Roche) at the following densities: VM-CUB-1 (2500 cells/well); 5637, CAL-29, HT-1376, UM-UC-6, UM-UC-10, T24, TCC-SUP-G, J82, and RT-112 cells (all at 5000 cells/well); UM-UC-3 (7500 cells/well); and JON (10,000 cells/well). The system recorded cell indices at 15 min intervals.

### 2.5. Cell Migration and Invasion Assay

Cell migration and invasion were determined using the xCELLigence RTCA system (Roche). Cells were transfected with the indicated siRNAs for 48 h and starved in a medium without FCS 24 h before the start of the real-time recording. Cells were seeded in 16-well CIM plates (Roche) at the following densities: UM-UC-6 and UM-UC-10 (both at 50,000 cells/well); HT-1376, TCC-SUP-G, JON, and RT-112 (all at 60,000 cells/well); J82 (70,000 cells/well); and CAL-29 (80,000 cells/well). For the cell migration experiments, the membrane between the upper and lower chambers of the CIM plate was left uncoated, whereas for the cell invasion assays, it was coated with 20 µL of Matrigel (Corning (Glendale, AZ, USA)). Matrigel was diluted in a serum-free medium to a concentration of 400 µg/mL and allowed to solidify at 37 °C for 4 h before coating. FCS (10%) was used as a chemoattractant. Cell indices were recorded every 15 min.

### 2.6. Organoid Isolation

For the establishment of bladder organoids, the bladders of C57BL/6 (Ctrl), *Pten*^fl/fl^/*Trp*53^fl/fl^, *Pten*^fl/fl^/*Trp*53^fl/fl^/*Kmt9α*^fl/fl^, and *Pten*^fl/fl^/*Trp53*^fl/fl^/*Kmt9αN122A*^flKI/flKI^ mice were isolated. Tumor tissue was manually dissected, and a single-cell suspension was generated using the Tumor Dissociation Kit, mouse (Miltenyi Biotec (Bergisch Gladbach, Germany)), according to the manufacturer’s protocol. Single cells were resuspended in a solution containing growth factor-reduced Matrigel (Corning (Glendale, AZ, USA)) and Advanced DMEM-F12 medium (#12634010, Thermo Fisher Scientific (Waltham, MA, USA) in a 60:40 ratio. For each dome, approximately 1000 cells were seeded in a 50 µL drop of Matrigel/Advanced DMEM-F12 in 24-well plates. The Matrigel was allowed to solidify at 37 °C for 60 min and then covered with 1 mL of culture medium.

### 2.7. Organoid Culture and Size Assessment

Mouse bladder organoids were maintained in Advanced DMEM-F12 (Ref: 12634-010) supplemented with penicillin/streptomycin, glutamine, B27 Supplement 1× (Gibco #17564044, Thermo Fisher Scientific (Waltham, MA, USA), 100 µg/µL of FGF-10 (PeproTech Germany, Hamburg, Germany #100-26), 25 µg/µL of FGF-7 (Peprotech #100-19), 0.2 µM of A83-01 (PeproTech Germany, Hamburg, Germany #9094360), and 10 µM of Y-27632 (Selleck Chemicals GmbH (Cologne, Germany), #S1049). For the in vitro deletion of the floxed alleles, organoids were treated with Cre-GFP-expressing adenovirus (1 × 10^10^ pfu/mL, Vectorbiolabs, Malvern, PA, USA, #1700, Lot A0501T#11M18). GFP-positive cells were isolated by FACS on day 3 after infection and allowed to regrow as organoids. The organoids were subcultured in Matrigel every 5–7 days. Pictures of organoids were taken using the EVOS™ FL cell imaging system. To determine organoid size, the diameter was measured with ImageJ (version 2.0.0-rc-69/1.52p).

### 2.8. Preparation of Cell Extracts

Human BLCA cells were transfected with siRNA and harvested 3 days later. Cell pellets were washed twice with cold PBS, resuspended in 1× cell lysis buffer (Cell Signaling, Danvas, MA, USA, #9803) and incubated on ice for 10 min. After sonication for 1 min and centrifugation for 10 min (14,000× *g* at 4 °C), the protein lysate in the supernatant was transferred to a new tube. For cell extracts of organoids, 8 domes per sample were harvested. Extracts were prepared as described above. Cell extracts from patient tissues were extracted using the Minilys homogenizer (Bertin technologies, Montigny-le-Bretonneux, France) and RIPA buffer. Samples were disrupted for 15 s at top speed. Protein concentrations were determined with a Bradford assay.

### 2.9. Western Blot

For Western blot, the following antibodies were used: anti-KMT9α (#27630, lot 20062017, Schüle Lab, Freiburg, Germany, 1:500, rabbit), anti-KMT9β (#28358, lot 27022018, Schüle Lab, 1:500, rabbit), anti-PTEN (CST#9552, lot 3 08/2019, Cell Signaling, 1:500, rabbit), anti-TRP53 (#LIN-P956, lot 6065476, Linaris BIOZOL, Eching, Germany, 1:500, rabbit), anti-β-Actin (#A1978, lot 0000086304, Sigma, 1:10,000, mouse), anti-EGFR (#2232, lot 17, Cell Signaling, 1:1000, rabbit), anti-AKT1 (#2938, lot 4, Cell Signaling, 1:1000, rabbit), anti-GAPDH (#M20028M, lot 244888, Abmart, Berkeley Heights, NJ, USA, 1:5000, mouse), and anti-tubulin (alpha tubulin, #T6074, lot 024M4837, Merck KGaA (Darmstadt, Germany)., 1:10,000, mouse). Western blots were developed and quantified using an Amersham Imager 600 (GE Healthcare, Buckinghamshire, UK).

### 2.10. RNA Preparation and RNAseq

5637 and CAL-29 cells were cultured in the presence of siCtrl or siKMT9α for three days prior to harvesting. *Pten*/*Trp53* KO and *Pten*/*Trp53* KO/*Kmt9αN122A* KI cells were harvested without prior siRNA treatment. RNA was isolated using the RNeasy Plus Mini kit (QIAGEN GmbH, Hilden, Germany) essentially as described by the manufacturer. RNA samples were sequenced using the standard Illumina protocol generating raw sequence files (.fastq files) by Novogene. Reads were aligned to the hg19 build of the human or mm10 build of the mouse genome using STAR version 2.7.10b. The aligned reads were counted with the Homer 4.11 software with default settings (analyzeRepeats) and differentially expressed genes were identified using EdgeR 2.38.4. *p* values of <10^–6^ were considered as statistically significant. Heatmaps were generated using Morpheus (https://software.broadinstitute.org/morpheus/, accessed on 15 April 2024). Data are deposited under GSE255344.

### 2.11. RNA Preparation for Quantitative RT-PCR and Analysis

Cells were cultured in the presence of siCtrl or siKMT9α for 3 days prior to harvesting. *Pten*/*Trp53* KO and *Pten* /*Trp53* KO/*Kmt9αN122A* KI cells were harvested without prior siRNA treatment. To isolate RNA, the RNeasy Plus Mini kit (QIAGEN GmbH, Hilden, Germany) was used according to the manufacturer’s protocol. RNA concentrations were measured using a Nanodrop 2000c instrument. Quantitative RT–PCR was performed using the Abgene SYBR Green PCR kit (Thermo Fisher Scientific (Waltham, MA, USA)) according to the supplier’s protocol. POLR2A was used for normalization. The following primers were used for RNA from human cells: POLR2A: 5′-GCACCACGTCCAATGACAT-3′ and 5′-GTGCGGCTGCTTCCATAA-3′, N6AMT1: 5′-ACGTTTCTGCTTTTGGACGC-3′ and 5′-TCAGTGCACATGTACAAAGCC-3′, NEK7: 5′-TGTGACTACCCACCTCTTCCTT-3′ and 5′-TGGGGAGGTATGACTTGCAC-3′, EGFR: 5′-CCAACCAAGCTCTCTTGAGG-3′ and 5′-GCTTTCGGAGATGTTGCTTC-3′, CCN2: 5′-TACCAATGACAACGCCTCCT-3′ and 5′-TGGGAGTACGGATGCACTTT-3′, WEE1: 5′-ATGTGCGACAGACTCCTCAA-3′ and 5′-GGCTTCCATGTCTTCACCAC-3′, AKT1: 5′-TCCTCCTCAAGAATGATGGCA-3′ and 5′-GTGCGTTCGATGACAGTGGT-3′, BIRC2: 5′-GCAGACACATGCAGCTCGAA-3′ and 5′-TTCCCAACACCTCAAGCCAC-3′, TNF: 5′-CAGCCTCTTCTCCTTCCTGAT-3′ and 5′-GCCAGAGGGCTGATTAGAGA-3′, CDK6: 5′-AACAGATATCGATGAACTAGGCAA-3′ and 5′-GAAGTATGGGTGAGACAGGGC-3′, DAG1: 5′-GACTGGGAAAACCAGCTTGA-3′ and 5′-GCCGCTGATACCTTGATGAT-3′, ICAM1: 5′-CAGAGGTTGAACCCCACAGT-3′ and 5′-CCTCTGGCTTCGTCAGAATC-3′, ITGA6: 5′-TTGCTAAACCTTCCCAGGTG-3′ and 5′-GGCCACTGAATGTTCAAGGT-3′. The following primers were used for RNA from mouse organoids: *Pgk1*: 5′-TGGTGGAGACACTGCCACTT-3′ and 5′-ACCCCAGGAAGGACTTTACC-3′, *Cdk6*: 5′-TGCTCAACCCATCGAGAAGT and 5′-GTTGGATGGCAGGTGAGAGT-3′, *Wee1*: 5′-GATACACGGCAAACTCCCCA-3′ and 5′-TGGCTTCCATGTCTTCAGAGG-3′, *Itga6*: 5′-CAGCGAAGGCAAAAGTGGTT-3′ and 5′-GTTGCTGTGCCGAGGTTTTT-3′, *Dag1*: 5′-TGCACTCAGTTCTCTCCGAC-3′ and 5′-TCCGTTGGAATGCTCACTCG-3′, *Birc5*: 5′-ACCTCAAGAACTACCGCATCG-3′ and 5′-TCTCGGTAGGGCAGTGGATG-3′, *Cav1*: 5′-AGGGCAACATCTACAAGCCC-3′ and 5′-TCGTTGAGATGCTTGGGGTC-3′, *Thbs1*: 5′-GACTCGGGACCCATCTATGA-3′ and 5′-GCAAGAAGAGAGGCAAGGAA-3′.

### 2.12. MTT Assay

Cell proliferation was determined using the CellTiter 96^®®^ Non-Radioactive Cell Proliferation Assay kit (Promega, Madison, WI, USA) essentially as described by the manufacturer. CAL-29 (1000 cells/well), 5637 (550 cells/well), UM-UC-3 (700 cells/well), RT-112 (600 cells/well), and J82 cells (400 cells/well) were seeded in 96-well plates in the presence of DMSO, KMI169, or KMI169Ctrl at different concentrations and allowed to grow for seven days prior to MTT measurement. Cell culture medium containing DMSO or an inhibitor was refreshed on day 4. Calculation of the GI_50_ was conducted using a sigmoidal model in GraphPad Prism 6.0.

### 2.13. Growth of Xenograft Tumors in NOD/SCID Mice

pLenti6-miRNA Control (miRNA Ctrl) and pLenti6-miRNA KMT9α (miRNA KMT9α) plasmids were used to produce recombinant lentivirus to infect 5637 cells as previously described [51]. For tumor inoculation, 4 × 10^6^ cells infected with miRNA Ctrl or miRNA KMT9α-producing virus were injected into NOD/SCID mice. Cells were resuspended in 50 µL RPMI 1640 mixed with 50 µL Matrigel on ice and administered subcutaneously into the flank of each animal. Tumors were removed on day 35. The sample size was *n* = 8 mice in each group. All experiments were performed according to the German Animal Protection Law with permission from department 35 of the regional council, Freiburg.

### 2.14. Statistical Analysis

Data are represented as mean ± s.d. Significance was calculated by two-tailed Student *t* test. Statistical significance was set to *p* < 0.05 and is represented as follows: ***, *p* < 0.001; **, *p* < 0.01; *, *p* < 0.05; and ns, not significant. Sample sizes are indicated where appropriate.

## 3. Results

### 3.1. KMT9α Depletion Blocks Proliferation and Migration of MIBC Cell Lines

**Figure 1:**
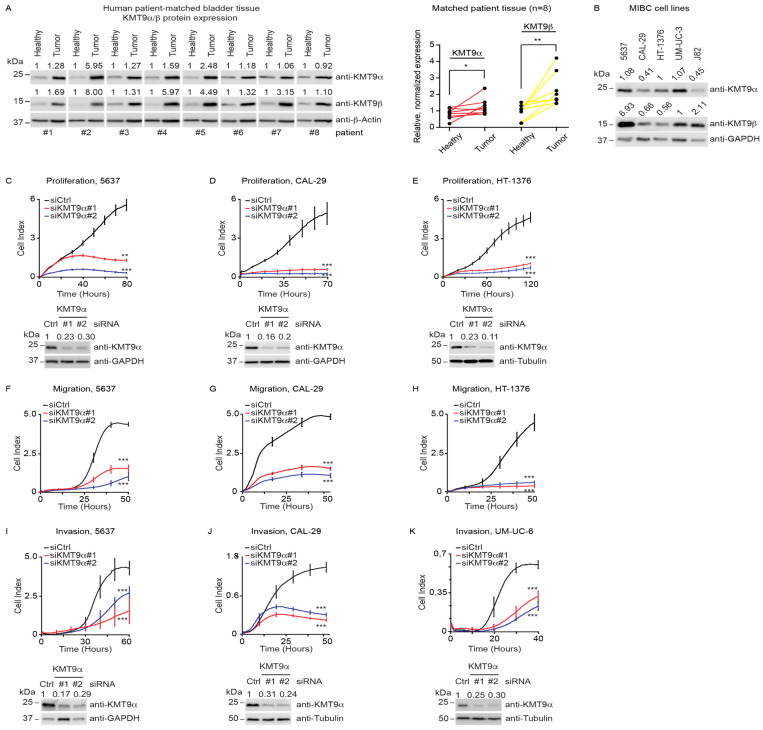
KMT9 is expressed in human MIBC tissue and cell lines and regulates proliferation, migration, and invasion. (**A**) Western blots of matched tumor and tumor-free tissue samples from patients with MIBC TNM stage T3a,b. Right panel: quantification of Western blot data. Statistical significance was calculated by Wilcoxon test (*, *p* < 0.05; **, *p* < 0.01). (**B**) KMT9α and KMT9β expression in human BC cell lines (5637, CAL-29, HT-1376, UM-UC-3, J82). (**C**–**E**) Real-time proliferation of (**C**) 5637, (**D**) CAL-29, and (**E**) HT-1376 cells after transfection with siCtrl, siKMT9α#1, or siKMT9α#2. (**F**–**H**) Real-time migration of (**F**) 5637, (**G**) CAL-29, and (**H**) HT-1376 cells after transfection with the above-mentioned siRNAs and overnight starving in serum-free medium. (**I**–**K**) Real-time invasion of (**I**) 5637, (**J**) CAL-29, and (**K**) UM-UC-6 cells after transfection with the above-mentioned siRNAs and overnight starving in serum-free medium. (**A**–**E**,**I**–**K**) Western blots were performed with the indicated antibodies. The intensity of each protein band was quantified by densitometry and normalized to the intensity of the house keeping gene. The median value determined for healthy tumor tissue (**A**) or all shown MIBC cell lines (**B**), and the siCtrl value (**C**–**E**,**I**–**K**) were set to 1. (**C**–**E**) Western blots represent the knockdown for proliferation (**C**–**E**) and migration assays (**F**–**H**) since the same batch of cells was used. (**C**–**K**) A representative experiment for each cell line is presented displaying the mean ± standard deviation derived from four technical replicates. (**C**–**K**) Statistical significance was calculated by two-tailed Student *t* test (**, *p* < 0.01; ***, *p* < 0.001). Each experiment was independently conducted at least three times. The knockdown efficiencies of KMT9α were confirmed by Western blot. (**A**–**E**,**I**–**K**) The original Western blots with molecular weight markers and original data of densitometry scans are presented in Figures S3 and S4.

To initiate our study, we assessed KMT9 expression in patient-derived MIBC tissue samples (TNM stages 3a,b) in comparison with patient-matched tumor-free tissue. The Western blot data revealed increases in KMT9α and KMT9β protein levels compared with patient-matched tumor-free tissue (Figure 1A). To explore the potential functions of KMT9 in MIBC, we investigated the effects of siRNA-mediated KMT9α knockdown in several MIBC cell lines (5637, CAL-29, HT-1376, UM-UC-3, and J82). KMT9α and KMT9β were expressed to various extents in all tested MIBC cell lines (Figure 1B). KMT9α was efficiently depleted with two different siRNAs (siKMT9α#1, siKMT9α#2) compared to a control siRNA (siCtrl) (Figure 1C–E,I–K and Figure S1A–I,Q,R).

To investigate the potential effects of KMT9α depletion on proliferation, migration, or invasion, we used the xCELLigence real-time cell analysis (RTCA) technology. The system continuously records changes in cell attachment to sensor electrodes at the bottom of plates used for proliferation assays, or at the underside of a separating membrane in plates used for migration/invasion assays [52]. Upon treatment with siKMT9α#1 or siKMT9α#2, we observed strongly the decreased proliferation of 5637, CAL-29, and HT-1376 cells compared with siCtrl-treated cells (Figure 1C–E). Similarly, the proliferation of various other human MIBC cell lines (UM-UC-6, UM-UC-10, J82, JON, TCCSUP, UM-UC-3, RT-112, T24, VM-CUB-1) was reduced (Figure S1A–I). Furthermore, KMT9α depletion affected migration (Figure 1F–H and Figure S1J–P) as well as invasion (Figure 1I–K and Figure S1Q–S) of numerous MIBC cells. Since the cell lines used harbour mutations in various genes such as *PTEN*, *TP53*, *PIK3CA*, *KRAS*, *HRAS*, *CDKN2A*, or *TERT* [53,54], our data suggest that KMT9 controls the proliferation and migration of BC cells regardless of the mutation profile.

### 3.2. Knockdown of KMT9α Affects Expression of Proliferation- and Migration-Related Genes

**Figure 2:**
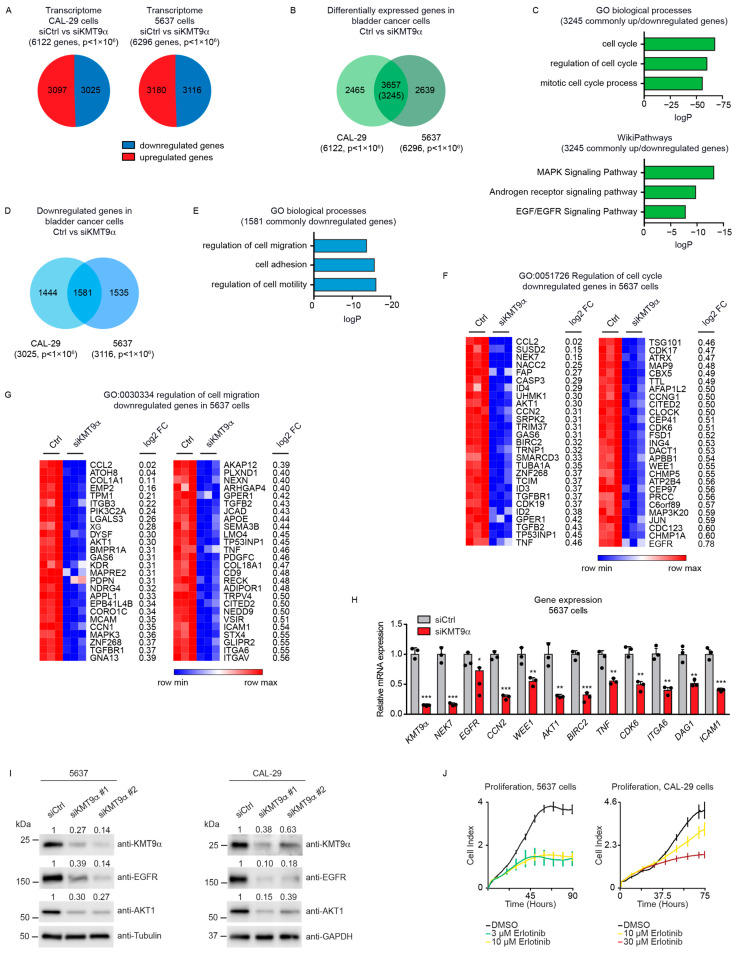
Knockdown of KMT9α affects expression of proliferation- and migration-related genes in MIBC cell lines. (**A**) Pie chart showing the number of differentially expressed genes (DEGs) in CAL-29 (6122) and 5637 (6296) cells upon siRNA-mediated knockdown of KMT9α (siKMT9α) with the number of upregulated and downregulated genes (*p* < 1 × 10^6^). Gene expression changes were determined in comparison with cells treated with a control siRNA (siCtrl). (**B**) Venn diagram showing overlap and number of DEGs (3657) in CAL-29 and 5637 cells upon knockdown of KMT9α (*p* < 1 × 10^6^). The value in brackets (3245) gives the number of DEGs that are deregulated in the same direction (up or down) in both cell lines. (**C**) Enriched gene sets for GO biological processes (top) and WikiPathways (bottom) obtained for the 3245 commonly deregulated genes. (**D**) Venn diagram showing overlap and number of downregulated genes (1581) in CAL-29 and 5637 cells upon knockdown of KMT9α (*p* < 1 × 10^6^). (**E**) Enriched gene sets for GO biological processes for these 1581 commonly downregulated genes. (**F**,**G**) Heat maps displaying relative mRNA levels of genes involved in (**F**) “regulation of cell cycle” (GO:0051726) or (**G**) “regulation of cell migration” (GO:0030334) in 5637 cells treated with siCtrl or siKMT9α. (**H**) Quantitative real-time PCR analysis of mRNA expression for selected genes presented in (**F**,**G**) after knockdown of KMT9α. Data represent means + standard deviation. Statistical significance was evaluated by a two-tailed *t* test (* *p* < 0.05; **, *p* < 0.01; ***, *p* < 0.001), *n* = 3. (**I**) Western blots showing protein expression of KMT9α, EGFR, and AKT1 in 5637 and CAL-29 cells after transfection with siCtrl, siKMT9α#1, or siKMT9α#2. Western blots were performed with the indicated antibodies. Tubulin or GAPDH served as controls. The intensity of each protein band was quantified by densitometry and normalized to the intensity of the house keeping gene. The siCtrl value was set to 1. (**J**) Real-time proliferation of 5637 cells (**left**) after treatment with DMSO, 3 µM, or 10 µM Erlotinib and CAL-29 cells (**right**) after treatment with DMSO, 10 µM, or 30 µM Erlotinib. (**I**) The original Western blots with molecular weight markers and original data of densitometry scans are presented in Figures S3 and S4.

To investigate gene expression changes upon KMT9α knockdown, we conducted a global transcriptome analysis (RNAseq) using CAL-29 and 5637 cells treated with siKMT9α#1 or siCtrl. We observed 6122 differentially expressed genes (DEGs) in CAL-29 cells and 6296 DEGs in 5637cells (Figure 2A). About half of these genes were upregulated and the other half downregulated. Intersection of the DEGs observed in both cell lines revealed 3657 common genes (Figure 2B), with the majority (3245 genes) deregulated in the same direction (up or down). Gene enrichment analyses for these 3245 commonly up- or downregulated DEGs identified biological processes including “regulation of cell cycle” and “mitotic cell cycle process” and WikiPathways such as the “MAPK signaling pathway” and “EGF/EGFR signaling pathway” among the top-ranking categories (Figure 2C). When specifically looking at the genes that were commonly downregulated upon KMT9α knockdown in both cell lines (1581 genes) (Figure 2D), the gene enrichment analysis revealed additional biological processes including “regulation of motility”, “cell adhesion”, and “regulation of cell migration” among the highest-ranked categories (Figure 2E). Heat maps for the genes listed in the term “regulation of cell cycle” are shown in Figure 2F (5637 cells) and Figure S2A (CAL-29 cells). The genes in the term “regulation of cell migration” are displayed as heat maps in Figure 2G (5637 cells) and Figure S2B (CAL-29 cells).

Differential expression of selected genes including *NEK7*, *EGFR*, *CCN2*, *WEE1*, *AKT1*, *BIRC2*, *TNF*, *CDK6*, *DAG1*, *ICAM1*, and *ITGA6* was validated by qRT-PCR analysis (Figure 2H and Figure S2C). Since EGFR is known to exert important functions in MIBC [55,56], we validated by Western blot that the protein levels of EGFR and AKT1 were reduced (Figure 2I). Furthermore, we observed that the effects on cell proliferation upon KMT9α knockdown were comparable to those elicited by the EGFR inhibitor Erlotinib (Figure 2J) [57]. Thus, the knockdown of KMT9α impairs the proliferation, migration, and invasion of human MIBC cell lines by the deregulation of gene sets mediating these processes. Altered EGFR and AKT expression may, at least in part, account for compromised MIBC cell proliferation and migration.

### 3.3. Cell Proliferation in Mouse Bladder Tumor Organoids Depends on the Catalytic Activity of KMT9

**Figure 3:**
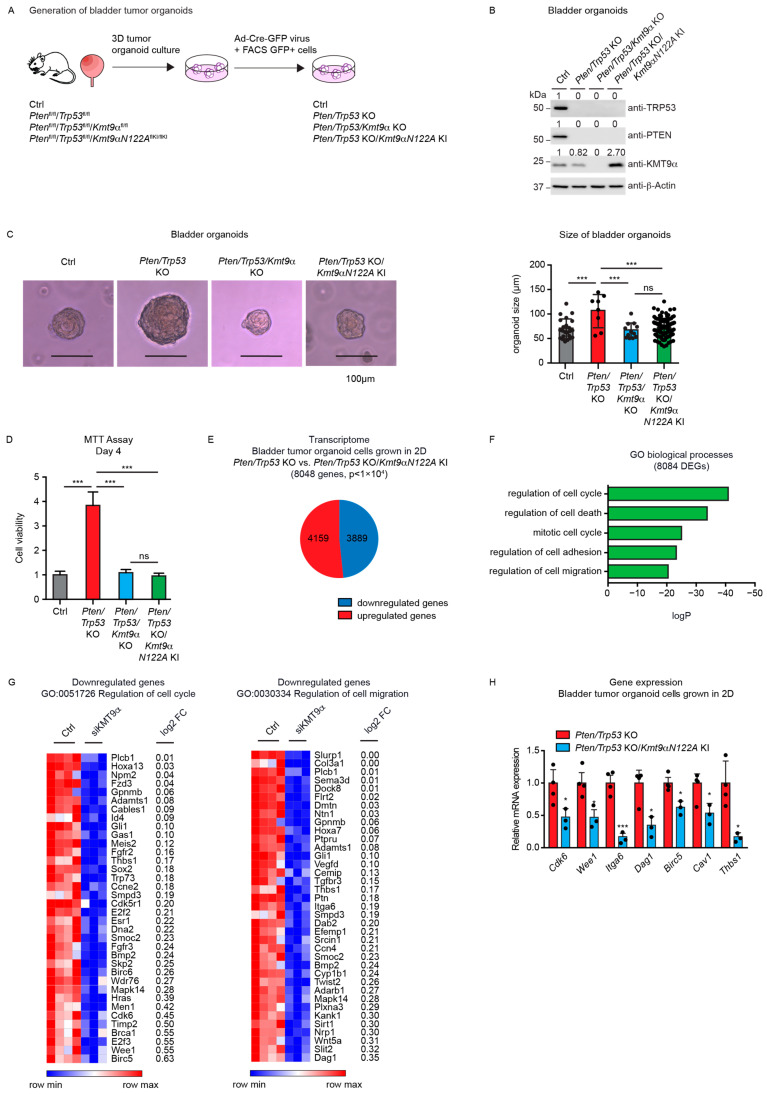
Cell proliferation in mouse bladder tumor organoids depends on the catalytic activity of KMT9. (**A**) Schematic of the establishment of bladder tumor organoids. Bladders of *Pten*^fl/fl^/*Trp*53^fl/fl^, *Pten*^fl/fl^/*Trp*53^fl/fl^/*Kmt9α*^fl/fl^, and *Pten*^fl/fl^/*Trp53*^fl/fl^/*Kmt9αN122A*^flKI/flKI^ mice were isolated, and single cells were grown under organoid culture conditions. Gene ablation was induced in vitro by infection of organoids with adenovirus driving Cre-GFP expression. GFP-positive cells isolated by Fluorescence-Activated Cell Sorting (FACS) were allowed to regrow as organoids. Organoids from C57BL/6 mice served as control. (**B**) Validation of depletion of PTEN, TRP53, and KMT9α by Western blot with the indicated antibodies. (**C**) Representative pictures (**left**) and relative size (**right**) of Ctrl, *Pten*/*Trp53* KO, *Pten*/*Trp53*/*Kmt9α* KO, and *Pten*/*Trp53* KO/*Kmt9αN122A* KI bladder (tumor) organoids 5 days after FACS. Scale bars, 100 μm. Statistical significance was evaluated by a one-way ANOVA test (***, *p* < 0.001; ns, not significant). (**D**) Relative cell viability of organoid cells grown as 2D cultures determined by MTT assay on day 4. Data represent means + s.d. Statistical significance was evaluated by a one-way ANOVA test (***, *p* < 0.001; ns, not significant), *n* = 3. (**E**) Pie chart showing the number of DEGs in *Pten*/*Trp53* KO compared with *Pten*/*Trp53* KO/*Kmt9αN122A* KI organoids (8048) with numbers of upregulated and downregulated genes (*p* < 1 × 10^4^). (**F**) Enriched gene sets for GO biological processes obtained for the 8048 DEGs. (**G**) Heat maps displaying relative mRNA levels of genes involved in “regulation of cell cycle” (GO:0051726) (**left**) and “regulation of cell migration” (GO:0030334) (**right**) in *Pten*/*Trp53* KO and *Pten*/*Trp53* KO/*Kmt9αN122A* KI cells. (**H**) Quantitative real-time PCR analysis of mRNA expression for selected genes presented in (**G**). Data represent means + standard deviation. Statistical significance was evaluated by a two-tailed *t* test, (*, *p* < 0.05; ***, *p* < 0.001) *n* = 3. (**B**) The original Western blots with molecular weight markers and original data of densitometry scans are presented in Figures S3 and S4.

Next, we investigated the potential effects of KMT9 ablation in tumor organoids derived from mice harboring alleles flanked with the following loxP sites: *Pten*^fl/fl^/*Trp53*^fl/fl^ (resulting in *Pten*/*Trp53* KO organoids after the Cre-induced deletion of alleles), *Pten*^fl/fl^/*Trp53*^fl/fl^/*Kmt9α*^fl/fl^ (resulting in *Pten*/*Trp53*/*Kmt9α* KO organoids), and *Pten*^fl/fl^/*Trp*53^fl/fl^/*Kmt9αN122A*^flKI/flKI^ (resulting in *Pten*/*Trp53* KO organoids with knock-in (KI) of the catalytically inactive mutant *KMT9αN122A*). We chose murine bladder organoids, which mainly consist of basal cells, since they better reflect in vivo organ functions compared with cell monolayers [40,41,42]. To generate organoids, single cells were isolated from mouse bladders and cultivated under appropriate growth conditions. Subsequently, gene ablation was induced in vitro by infection with Cre-GFP-expressing adenovirus. GFP-positive cells were isolated by Fluorescence-Activated Cell Sorting (FACS) and allowed to regrow as organoids (Figure 3A). As controls, we used organoids generated from C57BL/6 wildtype mice. Efficient gene deletion resulting in protein ablation was verified by Western blot (Figure 3B).

*Pten*/*Trp53* KO bladder tumor organoids were substantially larger than Ctrl wild-type organoids, suggesting an increased cell proliferation. Upon the ablation of *Kmt9α*, tumor organoid size was significantly decreased compared with the *Pten*/*Trp53* KO tumor organoids. Importantly, the knock-in of enzymatically inactive *KMT9αN122A* resulted in a comparable reduction in organoid size (Figure 3C), suggesting that the catalytic activity of KMT9 is required for the growth regulation of organoids. As the organoids could also be grown as adherent cells, we subsequently performed MTT assays using 2D cultures. In accordance with observations in 3D cultures, both *Kmt9α* knockout and the loss of catalytic activity impaired the viability of *Pten*/*Trp53* KO organoid cells grown as 2D cultures (Figure 3D).

We subsequently performed RNAseq with *Pten*/*Trp53* KO and *Pten*/*Trp53* KO/*KMT9αN122A* KI cells to better understand the effect of enzymatic inactivation on tumor cell growth. We observed 8048 DEGs, of which 4159 were upregulated and 3889 downregulated (Figure 3E). A gene enrichment analysis for these 8048 DEGs revealed biological processes such as “regulation of cell cycle”, “regulation of cell adhesion”, and “regulation of cell migration” among the top-ranking categories (Figure 3F). Heat maps for downregulated genes listed in the terms “regulation of cell cycle” and ”regulation of cell migration” are presented in Figure 3G. Using qRT-PCR, we confirmed expression changes in selected genes including *Cdk6*, *Wee1*, *Dag1*, *Itga6*, *Birc5*, *Cav1,* and *Thbs1,* the first four of which were also differentially expressed in 5637 and CAL-29 cells upon the knockdown of KMT9α (Figure 3H). Taken together, our data show that KMT9 regulates the growth of murine bladder tumor organoids, and that KMT9 enzymatic activity is required for growth control.

### 3.4. KMT9α Depletion Impairs BC Growth in Immunodeficient NOD SCID Mice

**Figure 4:**
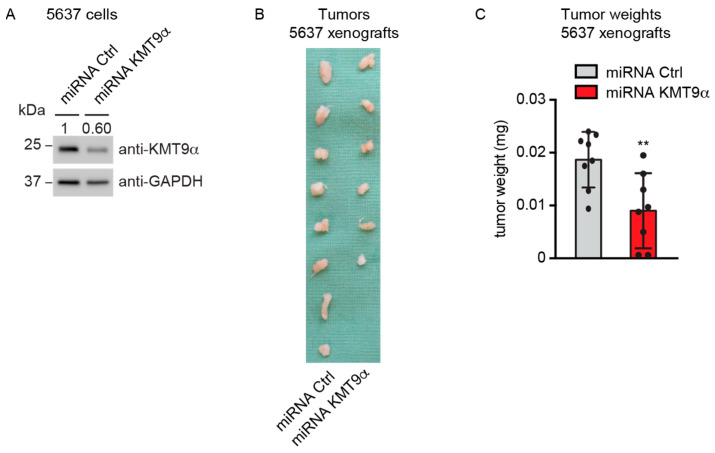
KMT9α depletion impairs BC growth in immunodeficient NOD SCID mice. (**A**) Western blot performed with the indicated antibodies to verify KMT9α knockdown efficiency in 5637 cells. Cells were infected with lentivirus driving expression of miRNA Ctrl or miRNA KMT9α. (**B**) Image of xenograft tumors and (**C**) tumor weight of 5637 cell xenografts isolated from mice on day 35, *n* = 8 mice per group. Data represent mean + s.d. Statistical significance was assessed by a two-tailed *t* test (**, *p* < 0.01). (**A**) The original Western blots with molecular weight markers and original data of densitometry scans are presented in Figures S3 and S4.

To explore whether KMT9α ablation also affected BC growth in vivo, 5637 cells were infected with lentivirus driving the expression of a microRNA targeting KMT9α (miRNA KMT9α) or a control microRNA (miRNA Ctrl). Western blot analysis validated the depletion of KMT9α protein (Figure 4A). Next, we subcutaneously implanted KMT9α-depleted or Ctrl 5637 cells into the flanks of immunocompromised mice. Notably, KMT9α depletion resulted in decreased growth and final weight of the xenografts compared with tumors in the control group (Figure 4B,C). Together, our results show that KMT9α depletion impairs the proliferation of multiple bladder cancer cell lines, *Pten*/*Trp53* KO tumor organoids, and xenografts in NOD SCID mice.

### 3.5. Inhibition of KMT9 Activity Blocks Proliferation of MIBC Cell Lines

**Figure 5:**
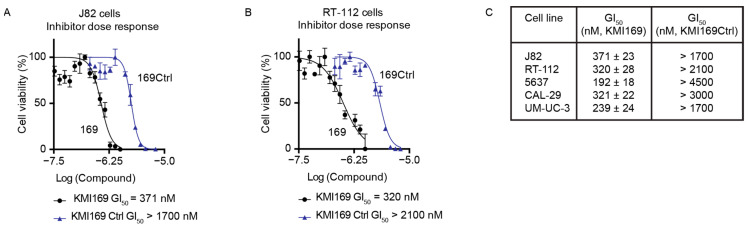
Inhibition of KMT9 activity blocks the proliferation of MIBC cell lines. (**A**–**C**) MTT viability assays were performed to determine the concentration of half-maximal growth inhibition (GI_50_) for KMI169 and KMI169Ctrl in (**A**) J82 and (**B**) RT-112 cells. (**C**) Table containing GI_50_ values for the inhibition of proliferation by KMI169 of different MIBC cell lines determined by MTT assays. Data represent means ± s.d. (*n* = 3).

Since MIBC cell proliferation depended on the catalytic activity, we finally investigated whether KMT9 inactivation using a small-molecule inhibitor also affected BC cell growth. Consequently, we treated different MIBC cell lines with our recently developed KMT9 inhibitor (KMI169) or the corresponding control compound (KMI169Ctrl) [50]. Compared with KMI169Ctrl, treatment with KMI169 strongly reduced the proliferation of various cell lines such as J82 cells with a half-maximal growth inhibition constant (GI_50_) of 371 nM and RT-112 cells with a GI_50_ of 320 nM (Figure 5A–C). KMT9 inhibition also impaired the proliferation of 5637, CAL-29, and UM-UC-3 cells (Figure 5C) [50]. These data provide evidence that KMT9 inhibition potently and selectively blocks the proliferation of MIBC cells and might be a novel therapeutic option for the treatment of this disease.

## 4. Discussion

In this study, we investigated the potential roles of KMT9 in MIBC. Our data show that KMT9α depletion impairs the proliferation, migration, and invasion of MIBC cell lines and the growth of murine *Pten*/*Trp53* KO tumor organoids as well as xenograft growth in mice. Since the KMT9-mediated control of cell growth depends on its enzymatic function, our data suggest that the inhibition of KMT9 enzymatic activity by small-molecule inhibitors might be a novel therapeutic strategy for MIBC. This idea is supported by the efficient growth inhibition of several MIBC cell lines by the KMT9 inhibitor KMI169.

The currently established medication for the treatment of advanced localized MIBC and metastatic urothelial carcinoma is a platinum-based systemic therapy [17,20]. In the case of platinum-ineligible or -refractory patients, the approved treatment alternatives include the PD-L1 inhibitors, Atezolizumab, Durvalumab, and Avelumab, as well as the PD-1 inhibitors, Pembrolizumab and Nivolumab. Additionally, Erdatifinib, a tyrosine kinase inhibitor targeting FGFR, gained approval for patients harboring FGFR2 or FGFR3 alterations [26,27]. Finally, Enfortumab vedotin and Sacituzumab govitecan, antibody–drug conjugates (ADC) that target Nectin-4 and Trop-2, were approved for patients who experienced progression after platinum-based chemotherapy [27,28,29]. While these therapies have significantly advanced the treatment of MIBC, their use is restricted to a narrow group of patients, and thus, novel methods of therapeutic intervention are required.

In light of our data, we propose that KMT9 might be a novel actionable target in MIBC. KMT9 is overexpressed in BC cancer samples compared with patient-matched adjacent tumor-free tissue. This result is in accordance with previous findings that the nuclear presence of KMT is increased in samples from patients with MIBC or metastasized urothelial carcinoma compared to normal urothelium and low-grade pTa samples [58]. Importantly, the observation that the small-molecule inhibitor KMI169 [50] impairs MIBC cell proliferation presents KMT9 inhibitors as promising candidates for novel therapeutic approaches. However, the evaluation of the therapeutic potential of KMT9 inhibitors requires their optimization to achieve more favorable absorption, distribution, metabolism, and excretion (ADME) properties for in vivo studies.

Another important area of BC research is the identification of biomarkers [11,12,13,14,15]. While several potential novel biomarkers in serum or urine samples have been proposed, clinically applicable biomarkers for BC diagnosis or therapy response prediction are currently either lacking or are underutilized [2,12]. In this study, we did not explicitly search for biomarkers. However, EGFR, which is downregulated upon KMT9 knockdown in 5637 and CAL-29 cells, has emerged as a prognostic factor in various cancer types, including BC. Elevated expression of EGFR in tumor tissue has been linked to diminished rates of recurrence-free survival or overall survival [59,60]. To date, we have only addressed the role of KMT9 in cellular BC functions. In future studies, it may be interesting to investigate the potential indirect roles of KMT9 on inflammatory processes (e.g., affecting the tumor microenvironment) or the urobiome, for example, via the deregulation of signaling molecules. This might help to address the question whether KMT9 functions in BC correlate with the occurrence of biomarkers or whether KMT9 might itself serve as a biomarker.

Former data have provided evidence that KMT9 plays a role in different types of cancer, where the ablation or inhibition of the enzyme affects tumor growth via distinct mechanisms including the deregulation of cell cycle progression and the induction of apoptosis (prostate cancer), non-apoptotic cell death (lung cancer), or the maintenance of stem cell populations (colorectal cancer) [44,48,49,50]. Thus, KMT9 appears to exert multiple functions depending on the exact molecular background of cancer cells. In addition to the regulation of cell proliferation, in human MIBC cells, the repertoire of KMT9-mediated control is extended by the modulation of cell migration and invasion.

Since differential gene expression upon KMT9α depletion affects biological processes such as “regulation of motility”, “cell adhesion”, and “regulation of cell migration”, our results indicate that KMT9 might play a role in metastasis. This hypothesis is supported by prominent DEGs including ICAM1, ITGA6, THBS1, CAV1, and BMP2, which are known to promote epithelial mesenchymal transition (EMT) and other processes involved in cell migration and invasion [61,62]. Aberrant expression of the integrin ITGA6, for example, was shown to affect cell–matrix interactions and cancer progression by the regulation of various signaling pathways such as PI3K/AKT or MEK/ERK [63,64,65,66,67]. Another interesting aspect of KMT9 function is the downregulation of EGFR upon KMT9α knockdown. EGFR is overexpressed in MIBC [55,56] and has been reported to be involved in numerous processes including cell proliferation, survival, and metastasis [68]. For example, crosstalk between EGFR and integrins has been observed, which influences cell motility and adhesion [69,70]. In prostate cancer cells, EGFR was shown to initiate EMT under the control of AKT [71]. Due to the prominent roles of EGFR signaling in BC, different drugs targeting EGFR have been recently tested in preclinical and clinical studies [72,73]. While EGFR inhibitors have shown limited effects in patients that were not pre-selected according to molecular subtype [72,73], other reports suggested that EGFR targeting might be particularly efficient in the basal/squamous cell carcinoma-like BC subtype [57]. It will be interesting to address in future studies the potential additive or cooperative effects of KMT9 and EGFR inhibition.

Metastasis and the failure of metastasis treatment are the most common causes of death of patients with solid tumors [74,75]. Accordingly, most patients who do not survive BC have also experienced symptomatic metastasis [76]. Therefore, it is of high interest to further investigate the potential involvement of KMT9 in metastatic processes as well as therapeutic implications. Since this study is primarily based on cell culture and organoid systems, it is necessary to include mouse metastasis models in future studies.

In summary, our study underlines the notion that targeting KMT9 could be a promising future therapeutic approach in advanced BC paving the way for further studies in this field.

## 5. Conclusions

KMT9 mediates the proliferation, migration, and invasion of various MIBC cell lines, regardless of their mutational profile, by regulating underlying processes. The growth of BC organoids relies on the enzymatic activity of KMT9. Accordingly, the small-molecule inhibitor KMI169 impairs MIBC cell proliferation. The findings of this study pave the way for the further evaluation of KMT9-targeted therapies in advanced BC. In the near future, the highest priorities should be given to the corroboration of KMT9 functions in BC mouse models and the optimization of ADME properties of inhibitors for in vivo studies.

## Data Availability

Dara are contained within the manuscript.

## Supplementary Materials

The following supporting information can be downloaded at: https://www.mdpi.com/article/10.3390/cancers16081532/s1, Figure S1: Knockdown of KMT9α blocks proliferation, migration, and invasion of MIBC cell lines. Figure S2: Knockdown of KMT9α induces gene expression changes in MIBC cell lines. Figures S3 and S4: Original Western blots.
