# Supplementary material for: Lysine Methyltransferase 9 (KMT9) Is an Actionable Target in Muscle-Invasive Bladder Cancer
Source: Cancers (Basel). 2024 Apr 17;16(8):1532. doi: 10.3390/cancers16081532 (PMC11049522; doi:10.3390/cancers16081532)
Supplement: Supplementary file 1 [file cancers-16-01532-s001.zip › cancers-2894106-supplementary.pdf]

Figure S1: Knockdown of KMT9α blocks proliferation, migration, and invasion of MIBC cell lines

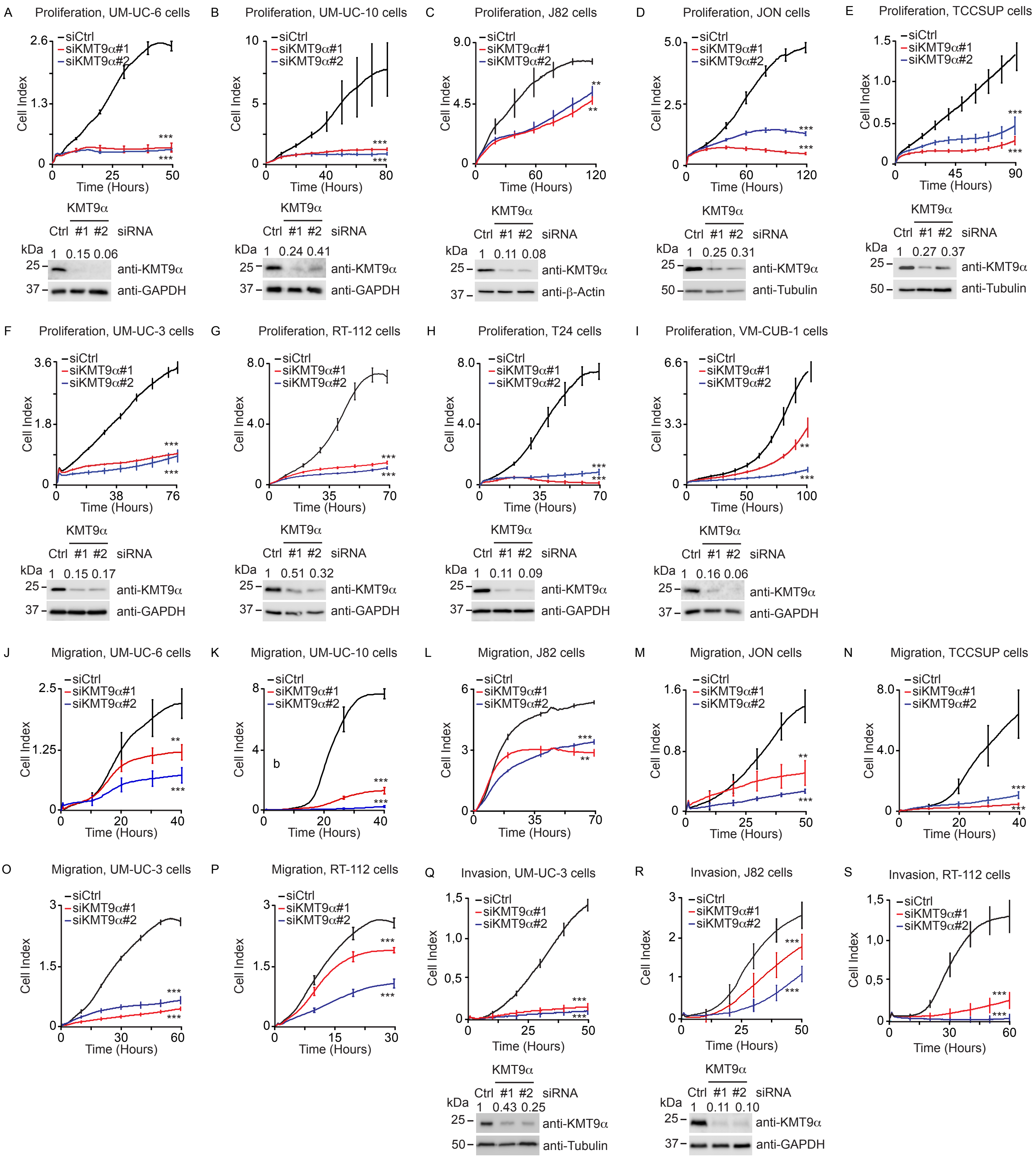

Figure S1. Knockdown of KMT9α blocks proliferation, migration and invasion of MIBC cell lines. (**A-I**) Real-time proliferation assays for (**A**) UM-UC-6, (**B**) UM-UC-10, (**C**) J82, (**D**) JON, (**E**) TCCSUP, (**F**) UM-UC-3, (**G**) RT-112, (**H**) T24 and (**I**) VM-CUB-1 cells after transfection with siCtrl, siKMT9α#1, or siKMT9α#2. (**J-P**) Real-time migration assays for (**J**) UM-UC-6, (**K**) UM-UC-10, (**L**) J82, (**M**) JON, (**N**) TCCSUP (**O**) UM-UC-3 and (**P**) RT-112 cells after transfection with the above-mentioned siRNAs and overnight starvation in serum-free medium. (**Q-S**) Real-time invasion assays for (**Q**) UM-UC-3, (**R**) J82 and (**S**) RT-112 cells after transfection with the above-mentioned siRNAs and overnight starvation in serum-free medium.(**A-S**) A representative experiment for each cell line is shown, displaying the mean ± standard deviation derived from four technical replicates. Each experiment was independently conducted at least three times. Statistical significance was calculated by two-tailed Student t test (**, $p < 0.01$; ***, $p < 0.001$). (**A-I,Q,R**) Western blot analyses using the indicated antibodies were done to confirm knockdown of KMT9α. The intensity of each protein band was quantified by densitometry and normalized to the intensity of the house keeping gene. The siCtrl value was set to 1. (**A-G**) Western blots represent the knockdown for proliferation (**A-G**) and migration assays (**J-P**) as well as the invasion assay of RT-112 cells (**S**) since the same batch of cells was used. (**A-I,Q,R**) The original Western blots with molecular weight markers and original data of densitometry scans are presented in Figures S3 and S4.

Figure S2: Knockdown of KMT9α induces gene expression changes in MIBC cell lines

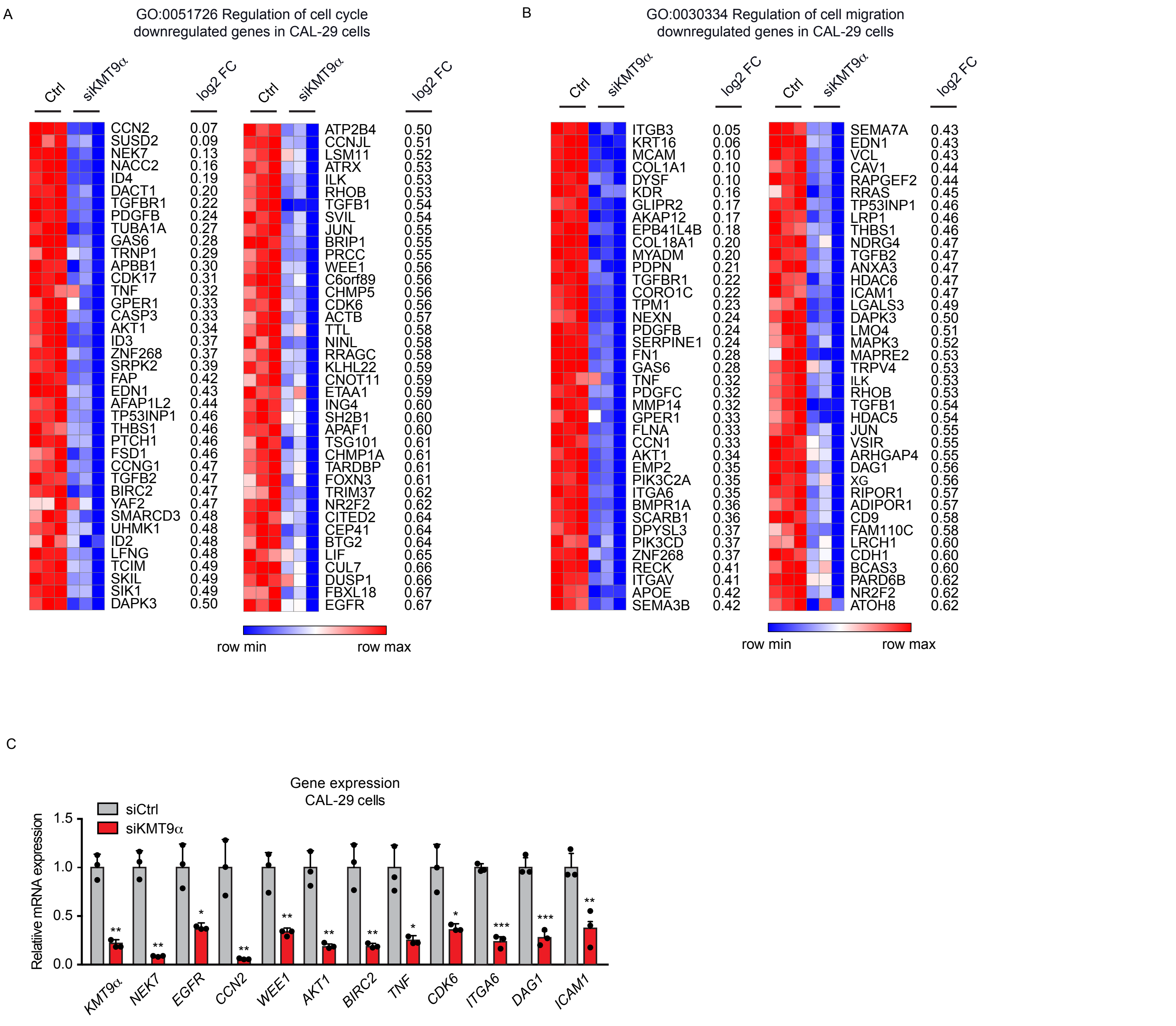

Figure S2. Knockdown of KMT9α induces gene expression changes in MIBC cell lines. (**A**) Heat map displaying relative mRNA levels of genes involved in "regulation of cell cycle" (GO:0051726) in CAL-29 cells treated with siCtrl or siKMT9α. (**B**) Heat map displaying relative mRNA levels of genes involved in "regulation of cell migration" (GO:0030334) in CAL-29 cells treated with siCtrl or siKMT9α. (**C**) Quantitative real-time PCR analysis of mRNA expression for selected genes presented in (**A**) and (**B**) after knockdown of KMT9α. Data represent means + standard deviation. Statistical significance was calculated by two-tailed Student t test (* $p < 0.05$, **, $p < 0.01$; ***, $p < 0.001$), $n = 3$.

Figure S3: Original Western blots

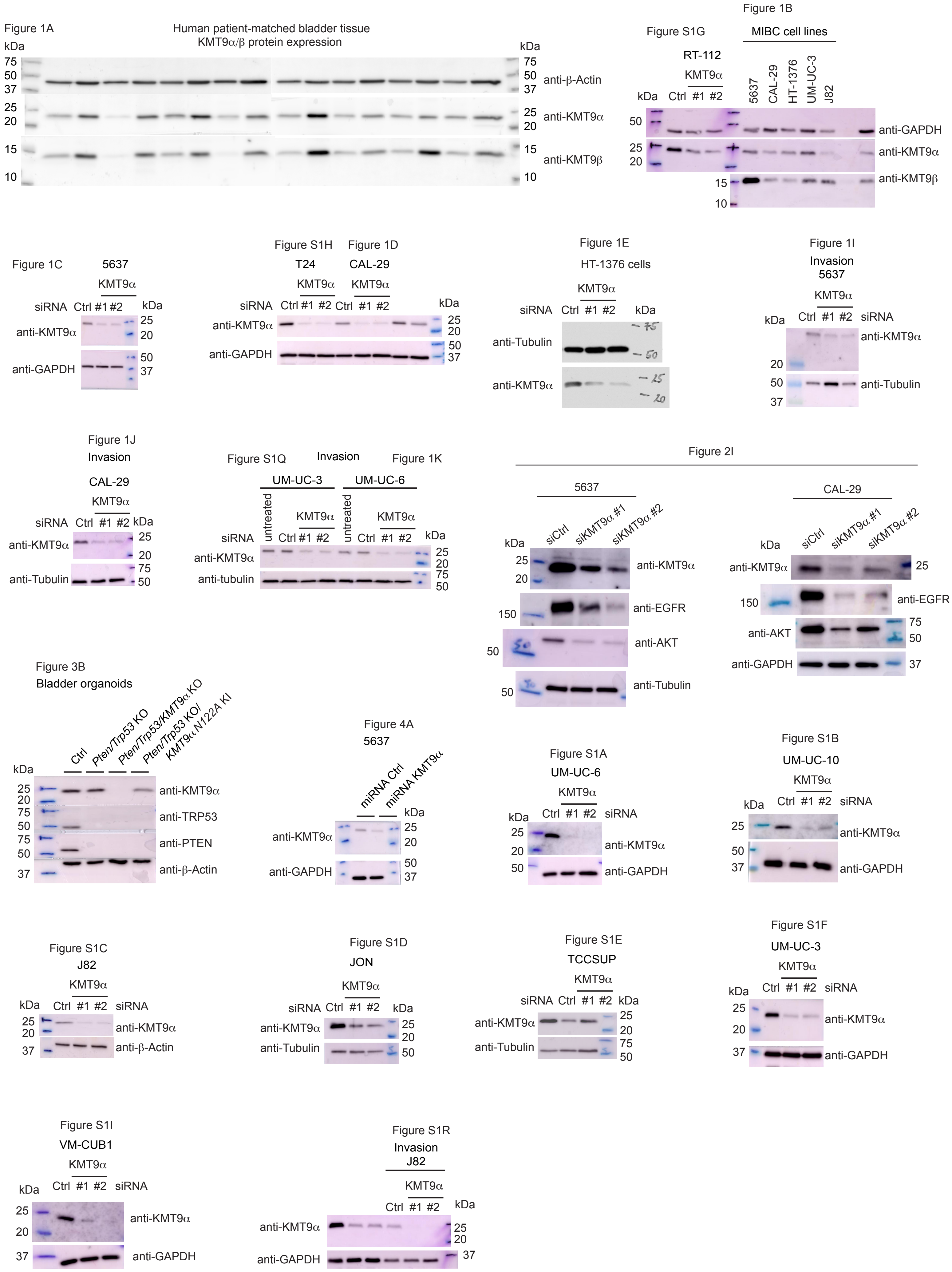

Figure S4: Original densitometry data

Figure 1A

Band intensity	#1 healthy	#1 tumor	#2 healthy	#2 tumor	#3 healthy	#3 tumor	#4 healthy	#4 tumor
β-Actin	46,822,614	62,228,340	44,319,426	51,067,269	44,880,536	62,592,516	51,026,506	77,247,789
KMT9α	24,857,561	42,428,975	5,781,084	39,645,192	31,187,000	55,200,847	17,094,149	41,186,843
KMT9β	46,822,614	62,228,340	44,319,426	51,067,269	44,880,536	62,592,516	51,026,506	77,247,789

Band intensity	#5 healthy	#5 tumor	#6 healthy	#6 tumor	#7 healthy	#7 tumor	#8 healthy	#8 tumor
β-Actin	42,651,732	50,631,010	43,901,706	62,094,837	44,095,897	53,853,668	38,456,748	80,636,822
KMT9α	23,602,812	69,457,543	17,305,402	28,912,022	21,337,852	27,703,346	25,423,824	49,255,319
KMT9β	16,011,577	85,314,782	19,482,296	36,309,837	19,323,544	74,282,778	21,990,933	54,528,678

Figure 1B

Band intensity	5637	CAL-29	HT-1376	UM-UC-3	J82
GAPDH	39,802,104	47,465,136	31,135,293	48,976,896	23,566,193
KMT9α	17,062,230	7,723,878	12,360,929	20,769,455	4,200,927
KMT9β	43,474,000	4,954,577	2,722,839	7,717,427	7,837,986

Figure 1C

Band intensity	siCtrl	siKMT9α #1	siKMT9α #2
KMT9α	1,596,187	385,704	415,541
GAPDH	19,214,006	19,791,369	16,593,005

Figure 1D

Band intensity	siCtrl	siKMT9α #1	siKMT9α #2
KMT9α	47,496,028	8,518,916	11,964,347
GAPDH	49633,557	54,951,858	63,598,368

Figure 1E

Band intensity	siCtrl	siKMT9α #1	siKMT9α #2
KMT9α	27,636,916	8,538,748	3,911,129
Tubulin	65,584,512	89,177,664	85,136,653

Figure 1I

Band intensity	siCtrl	siKMT9α #1	siKMT9α #2
KMT9α	7,817,037	3,324,063	2,238,016
GAPDH	4,678,613	11,717,871	4,560,766

Figure 1J

Band intensity	siCtrl	siKMT9α #1	siKMT9α #2
KMT9α	57,181,408	16,424,503	14,297,532
Tubulin	34,722,040	32,230,642	35,842,542

Figure 1K

Band intensity	siCtrl	siKMT9α #1	siKMT9α #2
KMT9α	10,306,471	2,680,510	2,462,317
Tubulin	41,364,780	43,336,209	33,310,376

Figure 2I (left)

Band intensity	siCtrl	siKMT9α #1	siKMT9α #2
Tubulin	48,953,399	47,101,267	44,894,550
KMT9α	61,793,754	16,171,794	7,975,738
EGFR	79,476,418	29,913,523	9,857,714
AKT1	37,002,053	10,664,857	9,030,927

Figure 2I (right)

Band intensity	siCtrl	siKMT9α #1	siKMT9α #2
GAPDH	29,199,794	33,375,034	31,766,324
KMT9α	13,017,508	5,714,838	8,856,652
EGFR	42,562,692	4,840,341	8,349,338
AKT1	9,861,695	1,744,134	4,237,722

Figure 3B

Band intensity	Ctrl	*Pten/Trp53* KO	*Pten/Trp53/ Kmt9α* KO	*Pten/Trp53* KO/ *Kmt9αN122A* KI
β-Actin	31,774,647	24,536,859	29,586,072	27,687,000
TRP53	7,376,589	0	0	0
PTEN	54,198,994	0	0	0
KMT9α	23,825,771	15,153,552	0	56,001,540

Figure 4A

Band intensity	miRNA Ctrl	miRNA KMT9α
KMT9α	64,782,872	29,881,665
GAPDH	68,332,363	52,927,940

Figure S1A

Band intensity	siCtrl	siKMT9α #1	siKMT9α #2
KMT9α	35,879,610	5,168,882	2,979,210
GAPDH	37,922,524	37,576,470	54,295,404

Figure S1B

Band intensity	siCtrl	siKMT9α #1	siKMT9α #2
KMT9α	46,670,931	9,494,632	17,580,147
GAPDH	57,661,179	48,072,360	52,878,086

Figure S1C

Band intensity	siCtrl	siKMT9α #1	siKMT9α #2
KMT9α	11,406,688	1,422,752	1,449,608
β-Actin	17,679,415	20,884,077	27,892,916

Figure S1D

Band intensity	siCtrl	siKMT9α #1	siKMT9α #2
KMT9α	49,179,177	16,254,502	28,983,339
Tubulin	15,158,877	19,965,719	28,703,748

Figure S1E

Band intensity	siCtrl	siKMT9α #1	siKMT9α #2
KMT9α	49,678,431	16,011,267	28,912,785
GAPDH	43,315,744	51,754,811	68,796,409

Figure S1F

Band intensity	siCtrl	siKMT9α #1	siKMT9α #2
KMT9α	53,372,167	8,662,005	10,208,604
GAPDH	53,172,647	56,044,237	60,045,620

Figure S1G

Band intensity	siCtrl	siKMT9α #1	siKMT9α #2
KMT9α	40,057,215	15,192,041	9,327,888
GAPDH	37,866,443	28,325,897	27,646,903

Figure S1H

Band intensity	siCtrl	siKMT9α #1	siKMT9α #2
KMT9α	91,437,333	10,272,474	8,412,143
GAPDH	53,584,869	55,769,147	56,175,640

Figure S1I

Band intensity	siCtrl	siKMT9α #1	siKMT9α #2
KMT9α	62,486,208	10,113,461	3,405,122
GAPDH	88,244,870	87,862,070	78,638,270

Figure S1Q

Band intensity	siCtrl	siKMT9α #1	siKMT9α #2
KMT9α	13,745,131	4,632,425	4,074,992
Tubulin	41,033,504	32,047,437	47,754,416

Figure S1R

Band intensity	siCtrl	siKMT9α #1	siKMT9α #2
KMT9α	24,118,568	2,594,596	2,861,270
GAPDH	44,253,751	44,273,708	52,697,727
